# Supplementary material for: Genomic Analysis of Antibiotic Resistance and Virulence Profiles in Escherichia coli Linked to Sternal Bursitis in Chickens: A One Health Perspective
Source: Vet Sci. 2025 Jul 17;12(7):675. doi: 10.3390/vetsci12070675 (PMC12298035; doi:10.3390/vetsci12070675)
Supplement: Supplementary file 1 [file vetsci-12-00675-s001.zip › vetsci-3715723-supplementary.pdf]

Figure S1: Distribution of virulence-associated genes were screened across *E. coli* isolates.

	JR25	JR26	JR29	JR30	JR32	JR34	JR35	JR37	JR39	JR42	JR43	JR46	JR48	JR50	JR52	JR53	JR54	JR56	JR58	JR60
fdeC	yes	yes	yes	yes	yes	yes	yes	yes	yes	yes	yes	yes	yes	yes	yes	yes	yes	no	yes	yes
fimA	yes	yes	yes	no	yes	yes	yes	yes	yes	no	yes	yes	yes	yes	yes	yes	yes	yes	yes	yes
fimB	yes	yes	yes	yes	yes	yes	yes	yes	yes	no	yes	yes	yes	yes	yes	yes	yes	yes	yes	yes
fimC	yes	yes	yes	yes	yes	yes	yes	yes	yes	no	yes	yes	yes	yes	yes	yes	yes	yes	yes	yes
fimD	yes	yes	yes	yes	yes	yes	yes	yes	yes	no	yes	yes	yes	yes	yes	yes	yes	yes	yes	yes
fimE	yes	yes	yes	yes	yes	yes	yes	yes	yes	no	yes	yes	yes	yes	yes	yes	yes	yes	yes	yes
fimF	yes	yes	yes	yes	yes	yes	yes	yes	yes	no	yes	yes	yes	yes	yes	yes	yes	yes	yes	yes
fimG	yes	yes	yes	yes	yes	yes	yes	yes	yes	no	yes	yes	yes	yes	yes	yes	yes	yes	yes	yes
fimH	yes	yes	yes	yes	yes	yes	yes	yes	yes	no	yes	yes	yes	yes	yes	yes	yes	yes	yes	yes
fimI	yes	yes	yes	yes	yes	yes	yes	yes	yes	no	yes	yes	yes	yes	yes	yes	yes	yes	yes	yes
ompA	yes	yes	yes	yes	yes	yes	yes	yes	yes	yes	yes	yes	yes	yes	yes	yes	yes	yes	yes	yes
yagV/ecpE	yes	yes	yes	yes	yes	no	no	no	no	no	no	no	yes	no	yes	yes	no	no	no	no
yagW/ecpD	no	no	no	no	no	yes	yes	yes	yes	yes	yes	yes	no	yes	no	no	yes	yes	yes	yes
yagX/ecpC	yes	yes	yes	yes	yes	yes	yes	yes	yes	yes	yes	yes	yes	yes	yes	yes	yes	yes	yes	yes
yagY/ecpB	yes	yes	yes	yes	yes	yes	yes	yes	yes	yes	yes	yes	yes	yes	yes	yes	yes	yes	yes	yes
yagZ/ecpA	yes	yes	yes	yes	yes	yes	yes	yes	yes	yes	yes	yes	yes	yes	yes	yes	yes	yes	yes	yes
ykgK/ecpR	yes	yes	yes	yes	yes	yes	yes	yes	yes	yes	yes	yes	yes	yes	yes	yes	yes	yes	yes	yes
gspC	yes	yes	yes	yes	yes	yes	yes	yes	yes	yes	yes	yes	yes	no	yes	yes	yes	no	yes	yes
gspD	yes	yes	yes	yes	no	yes	yes	yes	yes	yes	yes	yes	yes	no	yes	yes	yes	no	yes	yes
gspE	yes	yes	yes	yes	no	yes	yes	yes	yes	yes	yes	yes	yes	no	yes	yes	yes	no	yes	yes
gspF	yes	yes	yes	yes	no	yes	yes	yes	yes	yes	yes	yes	yes	no	yes	yes	yes	no	yes	yes
gspG	yes	yes	yes	yes	no	yes	yes	yes	yes	yes	yes	yes	yes	no	yes	yes	yes	no	yes	yes
gspH	yes	yes	yes	yes	no	yes	yes	yes	yes	yes	yes	yes	yes	no	yes	yes	yes	no	yes	yes
gspI	yes	yes	yes	yes	no	yes	yes	yes	yes	yes	yes	yes	yes	no	yes	yes	yes	no	yes	yes
gspJ	yes	yes	yes	yes	no	yes	yes	yes	yes	yes	yes	yes	yes	no	yes	yes	yes	no	yes	yes
gspK	yes	yes	yes	yes	no	yes	yes	yes	yes	yes	yes	yes	yes	no	yes	yes	yes	no	yes	yes
gspL	yes	yes	yes	yes	yes	yes	yes	yes	yes	yes	yes	yes	yes	no	yes	yes	yes	no	yes	yes
gspM	yes	yes	yes	yes	yes	yes	yes	yes	yes	yes	yes	yes	yes	yes	yes	yes	yes	no	yes	yes
hcp1/tssD1	yes	no	yes	yes	yes	yes	yes	yes	yes	no	yes	yes	yes	yes	no	yes	yes	yes	yes	yes
hcp2/tssD2	yes	no	yes	yes	yes	no	yes	yes	yes	no	yes	yes	yes	no	no	yes	yes	yes	no	yes
tssA	yes	no	yes	yes	no	no	yes	yes	yes	no	yes	yes	yes	yes	no	yes	yes	yes	no	yes
tssB	yes	no	yes	yes	yes	no	yes	yes	yes	no	yes	yes	yes	no	no	yes	yes	yes	no	yes
tssC	yes	no	yes	yes	no	no	yes	yes	yes	no	yes	yes	yes	no	no	yes	yes	yes	no	yes
tssF	yes	no	yes	yes	no	no	yes	yes	yes	no	yes	yes	yes	no	no	yes	yes	yes	no	yes
tssJ	no	no	yes	yes	no	no	yes	yes	yes	no	yes	yes	yes	no	no	yes	yes	yes	no	yes
tssL	no	no	yes	yes	no	no	yes	yes	yes	no	yes	yes	yes	no	no	yes	yes	yes	no	yes
tssM	yes	no	yes	yes	no	no	yes	yes	yes	no	yes	yes	yes	yes	no	yes	yes	yes	no	yes
vgrG/tssI	no	no	yes	yes	no	no	yes	yes	no	no	yes	no	yes	no	no	yes	yes	yes	no	yes
clpV/tssH	no	no	yes	yes	no	no	no	no	yes	no	yes	yes	yes	no	no	yes	yes	yes	no	yes
fha	no	no	yes	yes	no	no	yes	yes	yes	no	yes	yes	yes	no	no	yes	yes	yes	no	yes
gtrA	no	no	no	no	no	no	no	no	no	no	no	no	no	yes	no	no	no	no	no	no

kpsC	no	no	no	no	no	no	no	no	no	yes	no	no	no	no	no	no	no	no	no	no
kpsD	no	no	no	no	no	no	no	no	no	yes	no	no	no	no	no	no	no	no	no	no
kpsE	no	no	no	no	no	no	no	no	no	yes	no	no	no	no	no	no	no	no	no	no
kpsF	no	no	no	no	no	no	no	no	no	yes	no	no	no	no	no	no	no	no	no	no
kpsM	no	no	no	no	no	no	no	no	no	yes	no	no	no	no	no	no	no	no	no	no
kpsS	no	no	no	no	no	no	no	no	no	yes	no	no	no	no	no	no	no	no	no	no
kpsU	no	no	no	no	no	no	no	no	no	yes	no	no	no	no	no	no	no	no	no	no
manB	no	no	no	no	yes	no	yes	yes	no	no	no	no	no	no	no	no	no	yes	no	no
manC	no	no	no	no	yes	no	yes	yes	no	no	no	no	no	no	no	no	no	yes	no	no
entB	yes	yes	yes	yes	yes	yes	yes	yes	yes	yes	yes	yes	yes	yes	yes	yes	yes	yes	yes	yes
entC	yes	yes	yes	yes	yes	yes	yes	yes	yes	yes	yes	yes	yes	yes	yes	yes	yes	yes	yes	yes
entD	yes	yes	yes	yes	yes	yes	yes	yes	yes	yes	yes	yes	yes	yes	yes	yes	yes	yes	yes	yes
entE	yes	yes	yes	yes	yes	yes	yes	yes	yes	yes	yes	yes	yes	yes	yes	yes	yes	yes	yes	yes
entF	yes	yes	yes	yes	yes	yes	yes	yes	yes	yes	yes	yes	yes	yes	yes	yes	yes	yes	yes	yes
entS	yes	yes	yes	yes	yes	yes	yes	yes	yes	yes	yes	yes	yes	yes	yes	yes	yes	yes	yes	yes
fepA	yes	yes	yes	yes	yes	yes	yes	yes	yes	yes	yes	yes	yes	yes	yes	yes	yes	yes	yes	yes
fepB	yes	yes	yes	yes	yes	yes	yes	yes	yes	yes	yes	yes	yes	yes	yes	yes	yes	yes	yes	yes
fepC	yes	yes	yes	yes	yes	yes	yes	yes	yes	yes	yes	yes	yes	yes	yes	yes	yes	yes	yes	yes
fepD	yes	yes	yes	yes	yes	yes	yes	yes	yes	yes	yes	yes	yes	yes	yes	yes	yes	yes	yes	yes
fepG	yes	yes	yes	yes	yes	yes	yes	yes	yes	yes	yes	yes	yes	yes	yes	yes	yes	yes	yes	yes
fes	yes	yes	yes	yes	yes	yes	yes	yes	yes	yes	yes	yes	yes	yes	yes	yes	yes	yes	yes	yes
iroB	no	no	no	yes	no	no	yes	yes	no	yes	no	yes	yes	no	yes	yes	yes	no	no	yes
iroC	no	no	no	yes	no	no	yes	yes	no	yes	no	yes	yes	no	yes	yes	yes	no	no	yes
iroD	no	no	no	yes	no	no	yes	yes	no	yes	no	yes	yes	no	yes	yes	yes	no	no	yes
iroE	no	no	no	yes	no	no	yes	yes	no	yes	no	yes	yes	no	yes	yes	yes	no	no	yes
iroN	no	no	no	yes	no	no	yes	yes	no	yes	no	yes	yes	no	yes	yes	yes	no	no	yes
irp1	no	no	no	no	no	no	no	no	no	no	no	yes	no	no	yes	no	no	no	no	no
irp2	no	no	no	no	no	no	no	no	no	no	no	yes	no	no	yes	no	no	no	no	no
iucA	no	no	no	yes	no	no	no	no	no	yes	no	yes	yes	yes	yes	no	no	no	no	yes
iucB	no	no	no	yes	no	no	no	no	no	yes	no	yes	yes	yes	yes	no	no	no	no	yes
iucC	no	no	no	yes	no	no	no	no	no	yes	no	yes	yes	yes	yes	no	no	no	no	yes
iucD	no	no	no	yes	no	no	no	no	no	yes	no	yes	yes	yes	yes	no	no	no	no	yes
iutA	no	no	no	yes	no	no	no	no	no	yes	no	yes	yes	yes	yes	no	no	no	no	yes
fyuA	no	no	no	no	no	no	no	no	no	no	no	yes	no	no	yes	no	no	no	no	no
ybtQ	no	no	no	no	no	no	no	no	no	no	no	yes	no	no	yes	no	no	no	no	no
ybtE	no	no	no	no	no	no	no	no	no	no	no	yes	no	no	yes	no	no	no	no	no
ybtS	no	no	no	no	no	no	no	no	no	no	no	yes	no	no	yes	no	no	no	no	no
ybtX	no	no	no	no	no	no	no	no	no	no	no	yes	no	no	yes	no	no	no	no	no
ybtU	no	no	no	no	no	no	no	no	no	no	no	yes	no	no	yes	no	no	no	no	no
ybtA	no	no	no	no	no	no	no	no	no	no	no	yes	no	no	yes	no	no	no	no	no
ybtT	no	no	no	no	no	no	no	no	no	no	no	yes	no	no	yes	no	no	no	no	no
shuA	no	no	no	no	no	no	no	no	no	no	no	no	no	no	yes	no	no	no	no	no

chuW	no	no	no	no	no	no	no	no	no	no	no	no	no	no	yes	no	no	no	no	no
shuS	no	no	no	no	no	no	no	no	no	no	no	no	no	no	yes	no	no	no	no	no
chuT	no	no	no	no	no	no	no	no	no	no	no	no	no	no	yes	no	no	no	no	no
chuU	no	no	no	no	no	no	no	no	no	no	no	no	no	no	yes	no	no	no	no	no
shuV	no	no	no	no	no	no	no	no	no	no	no	no	no	no	yes	no	no	no	no	no
chuY	no	no	no	no	no	no	no	no	no	no	no	no	no	no	yes	no	no	no	no	no
shuX	no	no	no	no	no	no	no	no	no	no	no	no	no	no	yes	no	no	no	no	no
astA	yes	yes	yes	no	no	no	no	no	no	yes	no	no	no	yes	yes	no	no	no	no	yes
espL1	yes	yes	yes	yes	yes	yes	yes	yes	yes	yes	yes	yes	yes	yes	yes	yes	yes	yes	yes	yes
espL4	no	yes	no	no	no	no	no	no	no	no	no	no	no	yes	no	no	no	no	no	no
espR1	yes	yes	no	yes	yes	no	yes	yes	yes	yes	yes	yes	yes	yes	no	no	yes	yes	yes	yes
espX1	yes	no	no	yes	yes	yes	yes	yes	yes	yes	yes	yes	yes	yes	no	yes	yes	yes	yes	yes
espX2	no	yes	no	no	no	no	no	no	no	no	no	no	no	no	no	no	no	no	no	no
espX4	yes	no	yes	yes	yes	yes	yes	no	yes	yes	no	yes	yes	yes	no	yes	yes	yes	yes	yes
espX5	yes	yes	yes	yes	yes	yes	yes	yes	yes	yes	yes	yes	yes	yes	no	yes	yes	yes	yes	yes
espY1	no	no	no	no	no	no	no	no	no	no	no	no	no	yes	no	no	no	no	no	no
air	no	yes	no	no	no	no	no	no	no	no	no	no	no	no	no	no	no	no	no	no
anr	no	yes	no	yes	yes	no	yes	yes	no	yes	yes	yes	yes	yes	yes	no	no	no	yes	yes
AslA	no	yes	no	no	no	yes	no	no	no	yes	yes	no	no	yes	yes	no	no	no	yes	no
cea	no	no	no	no	no	no	no	no	no	no	no	no	no	no	yes	no	no	no	no	no
chuA	no	no	no	no	no	no	no	no	no	no	no	no	no	no	yes	no	no	no	no	no
cia	no	no	no	no	no	no	no	no	no	no	no	no	yes	no	no	yes	yes	no	no	no
cma	no	no	no	no	no	no	yes	yes	no	no	no	no	no	no	no	no	no	no	no	no
capU	no	no	no	no	no	no	no	no	no	yes	no	no	no	no	no	no	no	no	no	no
csgA	yes	yes	yes	yes	yes	yes	yes	yes	yes	yes	yes	yes	yes	yes	yes	yes	yes	yes	yes	yes
cvaC	no	no	no	yes	no	no	yes	yes	no	yes	no	yes	yes	no	yes	yes	yes	no	no	yes
eilA	no	yes	no	no	no	no	no	no	no	no	no	no	no	no	no	no	no	no	no	no
etpD	no	no	no	no	no	yes	no	no	no	no	no	no	no	no	no	no	no	no	yes	no
etsC	yes	no	no	yes	no	no	no	no	no	yes	no	yes	yes	no	yes	yes	yes	yes	no	yes
gad	yes	yes	yes	yes	yes	yes	yes	yes	yes	yes	yes	yes	yes	no	no	no	yes	yes	yes	yes
hha	yes	no	no	no	no	yes	no	no	no	no	no	no	no	no	no	yes	yes	yes	no	yes
hlyE	yes	yes	yes	yes	yes	yes	yes	yes	yes	yes	yes	yes	yes	yes	no	yes	yes	yes	yes	yes
hlyF	no	no	no	yes	yes	yes	yes	yes	no	yes	no	yes	yes	no	yes	yes	yes	no	yes	yes
hra	no	no	no	no	no	no	no	no	no	yes	no	no	no	yes	no	no	no	no	no	no
iha	no	no	no	no	no	no	no	no	no	no	no	no	no	yes	no	no	no	no	no	yes
ireA	no	no	no	no	no	no	no	no	no	yes	no	no	no	no	yes	no	no	no	no	no
iss	no	yes	yes	yes	yes	yes	yes	yes	yes	yes	no	yes	yes	yes	yes	yes	yes	no	yes	yes
lpfA	yes	no	no	yes	yes	no	yes	yes	yes	no	no	yes	yes	no	yes	yes	yes	yes	no	yes
mchB	no	no	no	no	no	no	no	no	no	no	no	no	no	no	yes	no	no	no	no	yes
mchC	no	no	no	no	no	no	no	no	no	no	no	no	no	no	yes	no	no	no	no	yes
mchF	no	yes	no	yes	no	no	no	no	no	yes	no	yes	yes	no	yes	yes	yes	no	no	yes
nlpI	yes	yes	yes	yes	yes	yes	yes	yes	yes	yes	yes	yes	yes	yes	yes	yes	yes	yes	yes	yes

ompT	yes	yes	yes	yes	yes	yes	yes	yes	yes	yes	no	yes	yes	no	yes	yes	yes	no	yes	yes
papC	no	no	no	no	no	no	no	no	no	no	no	no	no	no	yes	yes	yes	no	no	no
shiA	no	no	no	no	no	yes	no	no	no	yes	yes	no	no	yes	no	no	no	yes	yes	yes
shiB	no	no	no	no	no	no	no	no	no	yes	yes	no	no	yes	no	no	no	no	no	yes
sitA	no	no	no	yes	yes	yes	yes	yes	no	yes	yes	yes	yes	yes	yes	yes	yes	no	yes	yes
terC	yes	yes	yes	yes	yes	yes	yes	yes	yes	yes	yes	yes	yes	yes	yes	yes	yes	yes	yes	yes
tia	no	no	no	no	no	no	no	no	no	yes	yes	no	no	no	no	no	no	yes	no	no
traJ	yes	yes	yes	yes	yes	no	yes	yes	no	yes	yes	yes	yes	no	yes	yes	yes	no	no	yes
traT	yes	yes	yes	yes	yes	yes	yes	yes	no	yes	yes	yes	yes	no	yes	yes	yes	yes	yes	yes
tsh	no	no	no	yes	no	no	no	no	no	yes	no	yes	no	no	yes	no	no	no	no	yes
vat	no	no	no	no	no	no	no	no	no	no	no	no	no	no	yes	no	no	no	no	no
yehA	yes	yes	yes	yes	yes	yes	yes	yes	yes	yes	yes	yes	yes	yes	no	yes	yes	yes	yes	yes
yehB	yes	yes	yes	yes	yes	yes	yes	yes	yes	yes	yes	yes	yes	yes	yes	yes	yes	yes	yes	yes
yehC	yes	yes	yes	yes	yes	yes	yes	yes	yes	yes	yes	yes	yes	yes	yes	yes	yes	yes	yes	yes
yehD	yes	yes	yes	yes	yes	yes	yes	yes	yes	yes	yes	yes	yes	yes	yes	yes	yes	yes	yes	yes
